# Anthrax Toxin Uptake by Primary Immune Cells as Determined with a Lethal Factor-β-Lactamase Fusion Protein

**DOI:** 10.1371/journal.pone.0007946

**Published:** 2009-11-23

**Authors:** Haijing Hu, Stephen H. Leppla

**Affiliations:** Bacterial Toxins and Therapeutics Section, Laboratory of Bacterial Diseases, National Institute of Allergy and Infectious Diseases, National Institutes of Health, Bethesda, Maryland, United States of America; Columbia University, United States of America

## Abstract

**Background:**

To initiate infection, *Bacillus anthracis* needs to overcome the host innate immune system. Anthrax toxin, a major virulence factor of *B. anthracis*, impairs both the innate and adaptive immune systems and is important in the establishment of anthrax infections.

**Methodology/Principal Findings:**

To measure the ability of anthrax toxin to target immune cells, studies were performed using a fusion of the anthrax toxin lethal factor (LF) N-terminal domain (LFn, aa 1–254) with β-lactamase (LFnBLA). This protein reports on the ability of the anthrax toxin protective antigen (PA) to mediate LF delivery into cells. Primary immune cells prepared from mouse spleens were used in conjunction with flow cytometry to assess cleavage and resulting FRET disruption of a fluorescent β-lactamase substrate, CCF2/AM. In spleen cell suspensions, the macrophages, dendritic cells, and B cells showed about 75% FRET disruption of CCF2/AM due to cleavage by the PA–delivered LFnBLA. LFnBLA delivery into CD4+ and CD8+ T cells was lower, with 40% FRET disruption. When the analyses were done on purified samples of individual cell types, similar results were obtained, with T cells again having lower LFnBLA delivery than macrophages, dendritic cells, and B cells. Relative expression levels of the toxin receptors CMG2 and TEM8 on these cells were determined by real-time PCR. Expression of CMG2 was about 1.5-fold higher in CD8+ cells than in CD4+ and B cells, and 2.5-fold higher than in macrophages.

**Conclusions/Significance:**

Anthrax toxin entry and activity differs among immune cells. Macrophages, dendritic cells, and B cells displayed higher LFnBLA activity than CD4+ and CD8+ T cells in both spleen cell suspension and the purified samples of individual cell types. Expression of anthrax toxin receptor CMG2 is higher in CD4+ and CD8+ T cells, which is not correlated to the intracellular LFnBLA activity.

## Introduction

*Bacillus anthracis* is a gram-positive, spore forming bacterial pathogen. Anthrax infection occurs when dormant spores enter an animal and germinate, resulting in growth and dissemination of vegetative bacteria. Anthrax infections can occur in three forms, inhalational, gastrointestinal, and cutaneous, depending on the route by which spores enter the host [1]. Inhalational anthrax is the most severe form of infection, often leading to mortality unless rapidly diagnosed and treated.

After spore invasion, innate immunity is the front line of defense against infection. Early histopathological studies showed that in inhalational anthrax, spores in the lungs are efficiently taken up by phagocytic cells and transported to the regional lymph nodes [2]. There are contradictory reports on the fate of spores in macrophages. Some studies suggested that macrophages act as Trojan horses, allowing spores to germinate and grow into vegetative cells [3], [4]. Other researchers have shown that phagocytic cells can take up and kill spores or the newly germinated vegetative cells [5]–[7]. Mice depleted of macrophages are more susceptible to anthrax infection [8], supporting a protective role for these cells. Dendritic cells (DCs) are also capable of taking up spores [9] and lung DCs can transport spores to regional lymph nodes [10]. Human neutrophils can also engulf and effectively kill intracellular spores [11]. Neutrophil depletion in mice, however, does not alter the infection process [8].

To initiate infection, *B. anthracis* produces virulence factors to counter the host immune system. Anthrax toxin is an important virulence factor in the pathogenesis of anthrax. This three-part toxin consists of protective antigen (PA), edema factor (EF) and lethal factor (LF) [12]. PA (83 kDa) first binds to its cellular receptors, CMG2 and/or TEM8, and then furin or furin-like proteases cleave PA and release the N-terminal 20-kDa fragment [13]–[17]. The resulting 63-kDa species (PA63) assembles into an oligomeric complex having binding sites for LF and EF. After assembly of PA63 bound to EF (called edema toxin, ET) or LF (called lethal toxin, LT), the toxin complex enters cells by endocytosis. The low pH in endosomes triggers a conformational change in the complex which in turn allows LF/EF translocation to the cytosol of mammalian cells (for review see [18]). LF is a metalloproteinase that cleaves and inactivates the mitogen-activated protein kinase kinases (MEKs) 1–4, 6 and 7, and thereby blocks three pivotal mitogen-activated protein kinase (MAPK) pathways [19]–[21]. By targeting these important cell signaling pathways, LT suppresses cellular components of innate immunity. LT causes macrophages necrosis and apoptosis as well as blocking the production of pro-inflammatory cytokines and chemokines. LT also targets DCs, which bridge innate immunity and adaptive immunity. LT not only induces apoptosis of DCs so as to physically remove them from the system, but also blocks maturation of DCs so as to interrupt their ability to activate B cells and T cells [22]. The adaptive immune system is also attacked directed by LT, by blocking B cell proliferation and antibody production as well as T cell activation and proliferation [23]. EF is a calmodulin-dependent adenylate cyclase that elevates intracellular cAMP levels, thereby activating cAMP-dependent protein kinases and perturbing many cellular systems [24].

The small amounts of anthrax toxin that enter cells cannot easily be detected by direct visualization. Thus our lab previously developed a system utilizing a fusion protein, LFnBLA, consisting of the N-terminal region of LF (LFn) and β-lactamase (BLA), which together with the substrate coumarin cephalosporin fluorescein acetoxymethyl ester (CCF2/AM), allows imaging of the toxin delivery into cells [25]. CCF2/AM is a membrane permeable cephalosporin derivative that has fluorophores attached to opposite sides of the β-lactam ring of cephalosporin, resulting in fluorescence resonance energy transfer (FRET). The molecule normally shows an emission at 520 nm (green fluorescence) when excited at 409 nm. Cleavage of CCF2/AM by β-lactamase releases one fluorophore, disrupting the FRET, and shifting the emission to 440 nm (blue fluorescence). In this report we employed the LFnBLA system to assess the entry of LF into primary immune cells isolated from mouse spleens by measuring FRET disruption, allowing us to make predictions of the relative amounts of anthrax toxin entering various immune cells.

## Results

### Microscopic visualization of LFnBLA activity in spleen cells

**Figure 1:**
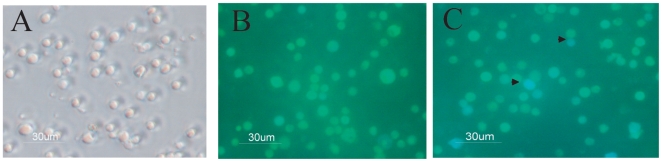
Fluorescence imaging of LFnBLA activity in spleen cells. Single cell suspensions were attached to polylysine-treated plates and then subjected to treatment with LFnBLA + PA followed by CCF2/AM staining. (A) Phase contrast photo showing the single cells in the spleen cell suspension. (B) Spleen cells treated with LFnBLA alone before CCF2/AM staining. (C) Spleen cells treated with LFnBLA + PA followed by CCF2/AM staining. The arrows point to cells with significant FRET disruption.

The spleen is an immunologic filter of the blood and contains macrophages, DCs, and B and T lymphocytes. Therefore we used mouse spleens as our source of immune cells. Although the β-lactamase activity of LFnBLA could be easily visualized in cell lines such as CHO cells and HeLa cells [25] (and data not shown), it was difficult to see significant differences between LFnBLA + PA treated and untreated spleen cells by microscopy after CCF2/AM staining (Figure 1). A Victor fluorescence reader (Perkin Elmer, Shelton, CT) also did not detect substantial differences in treated and untreated spleen cells (data not shown).

### Flow cytometric analyses of LFnBLA activity in spleen immune cells

**Figure 2:**
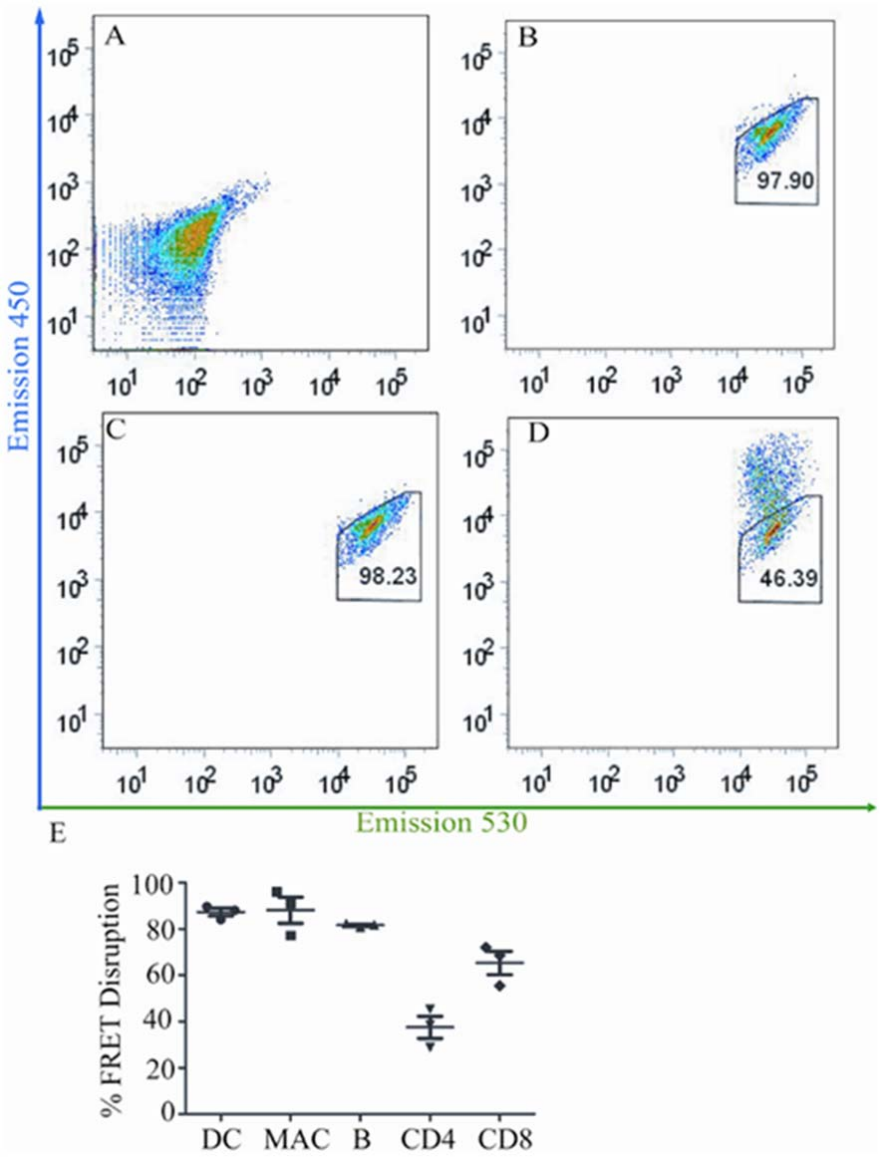
Flow cytometric analysis of LFnBLA activity in spleen cells. Spleen cells were treated with LFnBLA + PA and then stained with CCF2/AM. Cell specific markers were used to differentiate each immune cell type after CCF2/AM staining. (A) Unstained spleen cells showing autofluorescence. (B) Untreated spleen cells stained with CCF2/AM. (C) Spleen cells treated with LFnBLA alone followed by CCF2/AM staining. (D) Spleen cells treated with LFnBLA + PA followed by CCF2/AM staining. (E) Response of various types of immune cells to LFnBLA + PA treatment in a spleen cell suspension. Experiments were repeated three times, each time with a different mouse spleen, with error bars showing ±1 SD (standard deviation). (P = 0.008 comparing macrophages and CD8 cells).

We next examined the more sensitive flow cytometric method for its ability to detect LFnBLA activity. Single spleen cell suspensions were treated with LFnBLA + PA and stained with CCF2/AM before they were subjected to flow cytometry. We found that 54.6+/− 10.0% of spleen cells underwent FRET disruption after 1 h of LFnBLA + PA treatment, compared to the untreated control cells or those treated with LFnBLA alone (Figure 2B–2D). To further study the response of each type of immune cell to LFnBLA + PA treatment, cells were labeled with APC or PE-Cy7 conjugated antibodies specific for cell type-specific surface markers following CCF2/AM staining, so the FRET disruption of individual cell populations in the spleen cell suspension could be distinguished. DCs, macrophages and B cells showed about 75 to 80% FRET disruption, significantly greater than what was observed for both CD4+ and CD8+ T cells. CD8+ cells showed about 52% FRET disruption, and CD4+ cells had 42% FRET disruption (the lowest compared to the other four types of cells tested) (Figure 2E).

### Flow cytometric analyses of LFnBLA activity in purified cell populations

**Figure 3:**
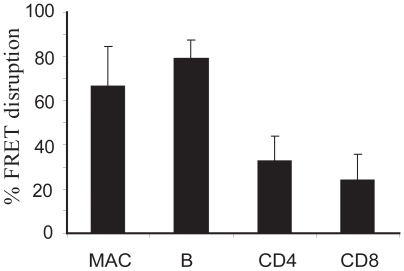
Flow cytometric analysis of LFnBLA activity in each type of immune cell from mouse spleens. Spleen cells were purified by magnetic beads and each type of cell was treated with LFnBLA + PA before CCF2/AM staining. Experiments were repeated three times. (P = 0.026 comparing macrophages and CD4 cells, P = 0.011 comparing B cells and CD4 cells, and P = 0.049 between CD4 and CD8 cells).

To test the activity of the LFnBLA fusion protein in purified immune cells, Miltyni microbeads were used to separate macrophages, B cells, CD4 cells and CD8 cells from the single spleen cell suspensions before LFnBLA/PA treatment. In purified immune cells, macrophages and B cells displayed about 65% and 80% of FRET disruption, respectively (Figure 3), higher than the activity detected in CD4+ and CD8+ T cells (32% and 25% of FRET disruption, respectively). This is similar to what was observed with the mixed spleen cell suspension. CD8+ cells, however, responded significantly higher than CD4+ cells in the mixed population (Figure 2), but responded similarly to CD4+ cells after purification. DCs indicated 86% FRET disruption, similar to macrophages and B cells (data not shown).

### Receptor expression level measurements by real-time PCR

**Figure 4:**
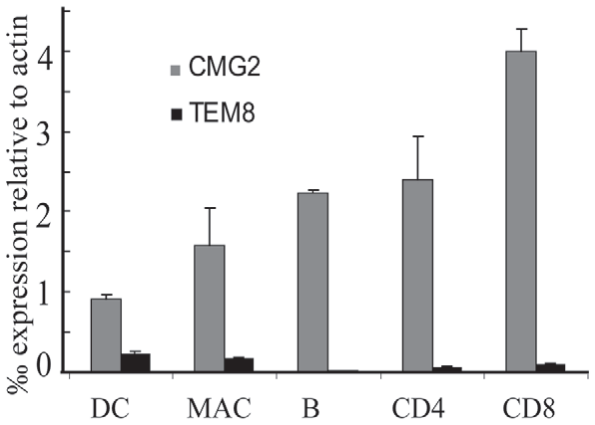
Real-time PCR result showing the relative expression levels of anthrax toxin receptors in immune cells. CMG2 and TEM8 expression levels in immune cells isolated from mouse spleens were normalized to those of β-actin in the same cell type. The data are reported as mean of amounts relative to β-actin ±1 SD.

To test if the enzymatic activity of the LFnBLA fusion protein in the cytosol was proportional to PA receptor levels expressed on immune cells, RT-PCR was performed to assess TEM8 and CMG2 levels in each cell population. Expression levels of TEM8 in all immune cells was much lower than that of CMG2, which recent evidence suggests is the more important and physiologically relevant receptor in mice [17]. Expression of CMG2 in CD8+ cells was about 1.5-fold higher than that in CD4+ and B cells, and 2.5-fold higher than in macrophages (Figure 4). DCs had the lowest CMG2 expression among all the immune cells isolated from mouse spleen, at about half the level in macrophages.

## Discussion

Anthrax toxin is important in the establishment of anthrax infection. Both LT and ET are produced very early after spore germination [26]. Toxin impairment of the host immune system allows vegetative bacteria to replicate, disseminate and eventually kill the host. It is important to determine the relative activities of the toxins in various immune cell types in order to better understand the interaction of this pathogen and the immune system. Direct measurement of the LF and EF proteins in cells is technically challenging, because receptor levels on most cell types average 20,000/cell [27]. Because the heptameric PA channel probably uses 7 receptor molecules to internalize at most 3 LF or EF molecules, and the process is unlikely to be error-free, it can be expected that cells on average internalize fewer than 10,000 molecules of LF or EF [25]. Furthermore, although the anthrax toxin receptors CMG2 and TEM8 are widely expressed in various animal tissues, the mouse spleen has a lower amount of receptors compared to the heart and liver [17]. While it may be possible to measure the consequences of LF and EF internalization (MEK cleavage and cAMP increases), these measurements each have features that make them inconvenient. In this study we used a fusion of the N-terminal domain of LF with β-lactamase to assess the ability of various cell types to internalize LF. β-lactamase fusions have been widely used as reporters to study bacterial and viral internalization by host cells [28], [29], and are also an effective tool to study the entry of bacterial effector proteins [30], [31]. The LFnBLA fusion protein presented in this work provides a useful tool to indirectly assess the entry/activity of toxin in various cells.

In this study, we titrated the concentrations of LFnBLA and PA in preliminary experiments and chose the lowest concentrations that consistently produced high FRET disruption. The selected concentration of 1 ug/ml PA (12 nM) is near the concentration needed to saturate the CMG2 and TEM8 receptors, which have apparent Kd values of about 1 and 10 nM, respectively [17]. These PA and LF concentrations were used because the objective was to assess the capacity of each cell type to internalize toxin, and it was therefore desirable to saturate the uptake system. These concentrations are probably not present in the early stages of an infection, but concentrations >200 ng/ml have been reported to occur late during an infection [32], [33].

To successfully establish an infection, *B. anthracis* needs to combat the host immune system. The innate response mounts a rapid defense after spores enter the host. Macrophages and DCs sequester the spores by phagocytosis. Previous research indicated that most intracellular spores germinate inside macrophages within the first hour [3], [7], but there are contradictory results on the fate of the germinated spores. Some reports show that spores grow into vegetative bacteria and escape from the macrophages, while other research suggests that macrophages efficiently kill germinated spores [4], [6], [7], [34]. DCs have been shown to be capable of transporting spores to regional lymph nodes [9], [10]. For the spores to initiate infection, they need to kill the phagocytic cells that sequester them soon after germination. Escape from phagocytic cells probably takes place before the adaptive immune response is initiated. Anthrax toxin is synthesized in the log phase after spores germinate and grow as vegetative cells [26], [35] and its accumulation following the germination of a few initial spores is required for other spores to escape phagocytic cells [36]. It would be consistent with this view if the DCs and macrophages were among the most toxin-sensitive immune cells, as they are the front line of the host defense, and sequestered spores escaping from these cells at the correct time is the most important step early in anthrax infection. In fact, our results show that macrophages, DCs and B cells have higher β-lactamase activity than T cells in both purified cells and mixed cell suspension, suggesting that these three cell types are more sensitive to anthrax LT.

In addition to countering innate immunity, *B. anthracis* also appears to target components of adaptive immunity through attacking B cells and antigen-presenting cells. DCs are the major antigen presenting cells that bridge the innate and adaptive immune systems [37]. DCs take up microbial pathogens, process them and display a specific portion of the pathogen on the surface to present to T cells. LT decreases DCs' ability to prime naïve T cells [38]. Since T cells can only recognize processed antigen in the context of the MHC, it may be that *B. anthracis* has evolved to efficiently impair the adaptive immunity by attacking DCs, which are fewer than 3% of the white blood cells in the spleen/blood, rather than attacking T cells directly.

The differences in LF activity towards various immune cell types observed in this work are not likely to be due only to differences in receptor levels. CMG2 binds to PA with up to 10-fold higher affinity than TEM8, and is the principal receptor contributing to in vivo toxicity of anthrax toxin [17]. We show here that expression of CMG2 is much higher than TEM8 in all types of the spleen immune cells tested, and the expression levels of CMG2 do not correlate with the intracellular β-lactamase activity observed. Macrophages, DCs and B cells, which exhibit the highest enzymatic activity, actually have lower amounts of receptor expressed. The internalization of LFnBLA (and therefore LF) to the cytosol involves a number of steps following receptor binding, including furin cleavage of PA, migration to lipid rafts, endocytosis, trafficking through endocytic vesicles, and translocation across membranes [39]. Each of these processes could differ in efficiency between the cell types studied here. Thus, it is not surprising that no correlation was observed between the expression levels of the major receptor CMG2 and the intracellular β-lactamase activity.

The use of LFnBLA for monitoring LF activity is not only important in studying disease initiation, but may facilitate cancer treatment in the future. Anthrax toxin-based variants have been shown to have an antitumor effect for several types of tumor, such as melanoma, fibrosarcomas and lung carcinoma [40]. The uptake and stability of the toxins in targeted cells is pivotal for the success of treatment. The method described here can assess the potency of toxin treatments against cancer cells [41], [42] and may also potentially be adapted for use in animal studies in the future.

## Materials and Methods

### Bacterial strains and protein purification

The LFnBLA fusion protein was purified from *Escherichia coli* BL21(DE3) [25] and PA was purified from *B. anthracis* as described previously [43].

### Spleen cell isolation

Mouse spleens were obtained from C57BL/6J mice (Jackson Laboratory, Bar Harbor, ME). Single cell suspensions were obtained by mechanical disruption and passage through a 70-µm cell strainer (BD Biosciences, San Jose, CA). Red blood cells were lysed by incubating the single cell suspension in 0.83% KHCO3- 0.1% NH4Cl-0.01 M EDTA (pH 7.4), a hypotonic buffer, on ice for 10 min. After three washes with PBS (Invitrogen, Carlsbad, CA) cells were resuspended in DMEM (Dulbecco's Modified Eagle Medium without phenol red, Invitrogen) and counted using a Cellometer (Nexcelom, Lawrence, MA).

### Spleen cell labeling and separation

Single spleen cell suspensions (10^8^ cells) from multiple mouse spleens were incubated in 1 ml blocking buffer (PBS, pH 7.4, 0.5% BSA, 2 mM EDTA). Magnetic beads conjugated with the cell-type specific markers CD11b, CD4 (L3T4), B220, or CD8 (Ly-2) (Miltenyi, Auburn, CA) were used to purify individual cell types using the protocols suggested by the manufacturer. Sorted cells were subjected to fluorescent labeling to check for purity by flow cytometry. Macrophages were >80% pure, while purities of other cell types were >90%.

### Microscopic visualization of LFnBLA activity

Tissue culture-treated, clear-bottom black-wall 96-well plates (Corning Inc., Corning, NY) were treated for 15 min with 0.1 mg/ml polylysine (50 µl/well) (Sigma Chemical Co., St. Louis, MO) prepared in PBS. After removal of polylysine, wells were washed with 100 µl PBS three times. Spleen cells were added (10^5^/well, suspended in DMEM + 2% FBS) and then treated with LFnBLA (2 µg/ml) plus PA (1 µg/ml) at 37°C for 1 h. Cells were then washed three times with PBS before adding Alternative Substrate Loading Solution (6 µl solution A containing CCF2/AM, 60 µl solution B, 925 µl solution C, and 2 mM probenecid). CCF2/AM and loading solution components were purchased from Invitrogen and probenecid was purchased from MP Biomedicals Inc. (Solon, OH). Cells were incubated with CCF2/AM at room temperature, avoiding light, for 4 h. Microscopy was performed using a Nikon TE2000-U Eclipse fluorescence microscope with BV-2A filter (excitation at 409 nm and emission at 447/520 nm) (Nikon, Melville, NY).

### Flow cytometric analysis of LFnBLA activity

Spleen cells were resuspended at 10^6^ per 0.5 ml in DMEM + 2% FBS, and treated with LFnBLA (2 µg/ml) plus PA (1 µg/ml) at 37°C for 1 h. After three washes with PBS, the cells were resuspended in 500 µl DMEM, and 100 µl modified loading solution (6 µl solution A, 60 µl solution B, 904 µl PBS and 0.1 mM probenecid) was added to cells. After 4 h of incubation at room temperature, cells were washed three times with PBS/probenecid (PBS supplemented with 0.1 mM probenecid). Cells were then resuspended in PBS/probenecid before flow cytometric analyses. To examine LFnBLA activity in individual cell types, after the CCF2/AM incubation and washes, spleen cell suspensions were stained with APC-anti-mouse CD11b, PE-Cy7-anti-mouse CD11c, PE-Cy7-anti-mouse B220, APC-anti-mouse CD4, or PE-Cy7-CD8a (BD Pharmingen, San Jose, CA). Antibodies were titrated to identify optimum concentrations. Staining was performed in PBS/probenecid with the optimized dilution of the labeled antibodies at 4°C for 20 min, followed by three washes with PBS/probenecid. The BD LSRII flow cytometer (BD Biosciences, San Jose, CA) was used to detect CCF2/AM FRET disruption by assessing emission at 440/40 nm for blue light and 525/50 nm for green light using excitation at 405 nm. The same method was used to treat the purified individual immune cells. Briefly, cells were resuspended with DMEM + 2% FBS after purification by Miltenyi magnetic beads, and treated with the same concentration of LFnBLA/PA. After three washes with PBS, the cells were stained with CCF2/AM with modified substrate loading solution and subjected to flow cytometric analysis. Flowjo software (TreeStar Inc., Ashland, OR) was used for data analysis.

### Real-time PCR

Total RNA isolated from purified mouse spleen immune cell populations using Trizol (Invitrogen) was subjected to reverse transcription to generate cDNA with the SuperScript II Reverse Transcriptase kit (Invitrogen). Real-time PCR analyses were carried out using the Applied Biosystems 7000 sequence detection system and the TaqMan gene expression master mix (Applied Biosystems, Foster City, CA). Primers for detection of CMG2 (Cat. No. Mm01196014_g1), TEM8 (Mm00712952_m1), and β-actin (Mm00607939_s1) were purchased from Applied Biosystems. TEM8 and CMG2 expression levels are presented as amounts relative to those of β-actin in each cell type.

### Statistical analysis

Student t-test analyses were performed using Graphpad Prism version 5.00 for Windows (GraphPad Software, San Diego California USA, www.graphpad.com).
